# Structural Properties of Lotus Seed Starch Nanocrystals Prepared Using Ultrasonic-Assisted Acid Hydrolysis

**DOI:** 10.3390/foods12102050

**Published:** 2023-05-19

**Authors:** Ru Jia, Minli Huang, Muhua Zeng, Sidi Liu, Wenjing Chen, Zebin Guo

**Affiliations:** 1 College of Food Science, Fujian Agriculture and Forestry University, Fuzhou 350002, China; 2 Fujian Provincial Key Laboratory of Quality Science and Processing Technology in Special Starch, Fujian Agriculture and Forestry University, Fuzhou 350002, China; 3 China-Ireland International Cooperation Centre for Food Material Science and Structure Design, Fujian Agriculture and Forestry University, Fuzhou 350002, China

**Keywords:** ultrasonic, acid hydrolysis, starch nanocrystals

## Abstract

This study provides a novel method of preparing lotus seed starch nanocrystals (LS-SNCs) using acid hydrolysis combined with ultrasonic-assisted acid hydrolysis (U-LS-SNCs) and evaluates the structural characteristics of starch nanocrystals using scanning electron microscopy; analysis of particle size, molecular weight, and X-ray diffraction patterns; and FT-IR spectroscopy. The results showed that the preparation time of U-LS-SNCs could be reduced to 2 days less than that for LS-SNCs. The smallest particle size and molecular weight were obtained after a 30 min treatment with 200 W of ultrasonic power and 5 days of acid hydrolysis. The particle size was 147 nm, the weight-average molecular weight was 3.42 × 10^4^ Da, and the number-average molecular weight was 1.59 × 10^4^ Da. When the applied ultrasonic power was 150 W for 30 min and acid hydrolysis was applied for 3 days, the highest relative crystallinity of the starch nanocrystals was 52.8%. The modified nanocrystals can be more widely used in various applications such as food-packaging materials, fillers, pharmaceuticals, etc.

## 1. Introduction

A lotus seed is the fruit or seed of the lotus (*Nelumbo nucifera* Gaertn.). It is mainly grown in Asia and has high nutritional value [1]. LSs also contain a large number of important industrial ingredients, such as vitamins, proteins, polyphenols, lipids, etc., which are beneficial for food formulation. The overall characteristics of lotus seeds make them a potential nutritional and bioactive food ingredient [2]. In China, lotus seeds have been reported to be included in 87 kinds of medicinal and edible food by the Ministry of Health [3]. The main component of lotus seeds is starch, making up about 50% of the weight of dried lotus seeds [4]. Lotus seed starch (LS) is an unusual kind of starch with amylose content as high as 42% [5]. In the study of corn starch, the crystallinity of corn starch decreases with the increase in amylose content [6]. The molecular structure of LS is characterized by a long main chain, a short branched chain, and a lesser degree of branching, rendering it easy to retrograde [7]. In addition, LS is insoluble in cold water and has a high viscosity and poor emulsifying ability after gelatinization in hot water, limiting its application in food processing [8].

Starch nanocrystals (SNCs) are nanosized crystals or semicrystals obtained after starch is modified to remove the amorphous region of starch particles [9]. Its advantages are its strong mechanical properties, high surface energy, and biological reproducibility, biocompatibility, and degradability that inorganic nanoparticles do not have [10]. At present, acid hydrolysis has become the principal method for preparing SNCs because it is easy to conduct and does not destroy the crystal structure of starch [11]. Angelier et al. [12] used a response surface methodology to explore the optimal process conditions for preparing waxy corn starch nanocrystals, namely, a sulfuric acid concentration of 3.16 M, hydrolysis for 5 days, an acidolysis temperature of 40 °C, and a stirring speed of 100 rpm. Quinoa starch nanocrystals were produced via acid hydrolysis; the highest yield was only 22.8% at 35 °C [13]. Although acid hydrolysis is a popular method for preparing starch nanocrystals, there are some issues associated with this method that hinder the industrial production and applicability of SNCs; namely, acid hydrolysis is time consuming, and the yield and recovery of SNCs are low [14,15].

Ultrasonic radiation consists of sound waves with a frequency range of 105–108 Hz, and these are elastic mechanical waves [16]. Ultrasonic modification is popular in the fields of food, dairy products, and medicine because of its mild, simple operation; low energy consumption; and low pollution [17]. Under the action of ultrasound, the accelerated movement of solvent molecules increases the friction between them and macromolecules that are difficult to move, which destroys the C–C bond of starch particles and, consequently, results in the degradation of their molecular chains, making it easier for acid to penetrate into the interior of starch particles and increasing the contact area between starch and acid [18]. Recently, the application of ultrasound treatment to produce nanocrytals from starch has been the subject of some studies. Shabana et al.’s [19] research results show that the physical and structural properties of starch nanoparticles prepared via ultrasonic-assisted acid hydrolysis are better than those prepared using acid hydrolysis or an ultrasonic method (a physical method). In another study, the simultaneous treatment of corn starch using ultrasound and sulfuric acid hydrolysis was investigated, focusing on a reduction in the starch nanocrystal preparation time while increasing the yield [14].

During the last decade, our research group has been engaged in basic theoretical and applied research on lotus-seed-starch (LS)-related topics. Through in-depth exploration of the relationship between the structure of LS and its gelatinization and aging mechanisms, our research group used processing techniques such as ultra-high pressure [4], microwave [20], ultrasound [20], and high-pressure homogenization [8] to provide a rich research foundation for inhibiting the aging of lotus seed products, improving the storage quality of lotus seeds, extending the shelf life of lotus seed products, and enhancing the nutritional value of lotus seeds. In terms of nano-research on lotus seed starch, Lin et al. [21] successfully prepared lotus seed starch nanoparticles via the enzymolysis of LS with Pullulanase. The crystalline structure of the starch particles changed from C type to B type, their crystallinity increased, and their particle size significantly decreased. The degradation of micron-sized LS particles to the nanoscale has expanded the application range of lotus seed starch to a certain extent, but it still fails to meet the needs regarding development in the food industry.

In the initial stage, our research team explored the conditions for preparing high-quality lotus seed starch nanocrystals (LS-SNCs) via acid hydrolysis (using a sulfuric acid concentration of 4 mol/L, a temperature of 40 °C, and hydrolysis for 5 days) [22]. On this basis, to further improve the structural characteristics of LS-SNCs and shorten the time for preparing LS-SNCs via acid hydrolysis, in this paper, LS was pretreated using ultrasonic radiation with different powers (100, 150, and 200 W), and then acid hydrolysis was carried out for 1, 3, and 5 days to prepare lotus seed starch nanocrystals (U-LS-SNCs). The effects of different factors on the structural and physical properties of starch nanocrystals were studied, and the optimal preparation process parameters were determined. Thus, this study provides a theoretical basis for the preparation of starch nanoparticles using the composite method.

## 2. Materials and Methods

### 2.1. Materials

Lotus seeds were purchased from Green Field Fujian Co., Ltd. (Fujian, China). Sulfuric acid (H_2_SO_4_), absolute ethanol, potassium bromide, and dimethyl sulfoxide (DMSO) were acquired from Sinopharm Chemical Reagent Co., Ltd. (Shanghai, China).

### 2.2. Sample Preparation

#### 2.2.1. Starch Extraction

Starch was extracted from lotus seeds according to the method reported by Sun et al. [23] with some modifications. Soaked lotus seeds were subjected to a high-speed tissue grinder and filtered with a 100-mesh sieve. Subsequently, the product was placed in a suspension for 6 h and washed with distilled water three times. Then, the suspension was washed with 90% ethanol three times. The defatted samples were dried at 45 °C and filtered with an 80-mesh sieve.

#### 2.2.2. Preparation of Lotus Seed Starch Nanocrystals (LS-SNCs)

This procedure was carried out according to the method reported by Hao et al. [24], with some modifications. A total of 15 g of LS was added to a conical flask containing 100 mL 4 mol/L H_2_SO_4_ solution; then, the conical flask was placed into a 40 °C water bath and reacted for 1, 3, and 5 days with a stirring speed of 200 r/min. After the reaction, the suspension was washed repeatedly with distilled water until reaching neutrality, and the centrifuged precipitate was freeze-dried to obtain a white powder of LS-SNCs.

#### 2.2.3. Preparation of Lotus Seed Starch Nanocrystals (U-LS-SNCs)

Treatment of LS via ultrasound was performed according to the method reported by Sujka and Jamroz [25] with a slight modification. A total of 30 g of LS was added to 100 mL of distilled water to produce a 30% (*m*/*v*) starch suspension. LS was treated with ultrasonic waves of different powers (100, 150, and 200 W) for 30 min. The ultrasonic-treated starch suspension was centrifuged at 3040× *g* for 10 min. The centrifuged sediment was dried in a 45 °C oven for 24 h and then ground through a 100-mesh sieve to obtain ultrasonic-treated LS, which was stored in a dryer for later use. The LS treated with different ultrasonic powers was treated according to the method reported in Section 2.2.2 to obtain the U-LS-SNCs.

### 2.3. Scanning Electron Microscopy (SEM)

This was procedure was performed according to the method reported by Zhao et al. [20]. The morphologies of LS-SNCs and U-LS-SNCs were examined using field emission SEM (FEI Nova Nano SEM 230; FEI Company, Hillsboro, OR, USA). In the low-vacuum mode, the acceleration voltage was 20 kV. The sample was sprayed onto an aluminum column with a conductive paste and coated with a thin film (50 nm) of gold.

### 2.4. Particle Size Measurement

Particle size measurements of LS-SNCs and U-LS-SNCs were performed at 25 °C using a laser particle size meter (Mastersizer 3000; Malvern Instruments Ltd., Malvern, Worcestershire, UK). The samples (0.01%, *m*/*v*) were suspended in ultrapure water. The refractive indices of the dispersant and sample were 1.33 and 1.53, respectively. The measurements were performed three times for each sample.

### 2.5. Molecular Weight (Mw) Distribution

The *Mw* distribution of LS-SNCs and U-LS-SNCs was obtained using gel permeation chromatography (Agilent PL-GPC220; Agilent Technologies Co., Ltd., Shropshire, UK). Ten-milligram samples of starch granules were dispersed in 10 mL of dimethyl sulfoxide (DMSO) at 100 °C for 2 h. The solution was continuously heated at 60 °C for 6 h with a magnetic stirrer. Then, the starch liquid was filtered through 0.45 μm nylon syringe filters before analysis. The dn/dc for calculation of the starch molecular weight was 0.072. The flow rate of the mobile phase was set to 0.5 mL/min.

### 2.6. X-ray Diffraction (XRD) Spectral Measurement

This step was performed with reference to Zhao et al.’s method with some modifications. [20]. XRD patterns of LS-SNCs and U-LS-SNCs were obtained using an X-ray diffractometer (RINT-TTR III; Rigaku Co., Tokyo, Japan). The Cu-Kα wavelength was 1.54056 Å, the scan tube voltage was 40 kV, the current was 200 mA, and the diffraction angle ranged from 5° to 35° (2θ). The data acquisition step width was 0.02°. The calculation of relative crystallinity followed the method reported by Dome et al. [26].

### 2.7. Fourier Transform Infrared (FT-IR) Spectral Measurement

FT-IR spectral measurements of LS-SNCs and U-LS-SNCs were obtained using a Fourier Transform infrared spectrometer (Tensor 27, Bruker, Karlsruhe, Germany). The relative ratio of crystalline and amorphous regions of the starch structure was expressed using an absorbance ratio of 995 cm^−1^/1022 cm^−1^ as a sensitive indicator of the short-range ordered structure of starch.

### 2.8. Statistical Analysis

All experiments were conducted in at least three parallel tests. Only SEM and *Mw* were recorded once. Origin Pro 8.5 software (OriginLab Corporation, Northampton, MA, USA) was used to create an experimental chart, and DPS 9.5 Software (Science Press, Beijing, China) was used to analyze the data.

## 3. Results and Discussion

### 3.1. Morphological Structure of LS-SNPs and U-LS-SNCs

**Figure 1:**
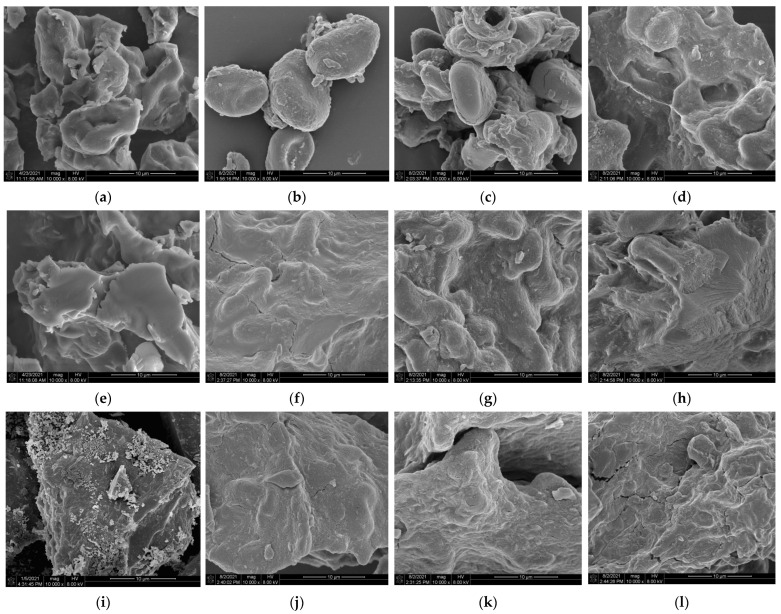
SEM images of LS-SNCs prepared using acid hydrolysis and U-LS-SNCs prepared using ultrasonically assisted acid hydrolysis. “W” and “d” represent ultrasonic power and acid hydrolysis days, respectively. U-LS-SNCs and LS-SNCs produced by employing (**a**) 0 W for 1 d, (**b**) 100 W for 1 d, (**c**) 150 W for 1 d, (**d**) 200 W for 1 d, (**e**) 0 W for 3 d, (**f**) 100 W for 3 d, (**g**) 150 W for 3 d, (**h**) 200 W for 3 d, (**i**) 0 W for 5 d, (**j**) 100 W for 5 d, (**k**) 150 W for 5 d, and (**l**) 200 W for 5 d.

The SEM results regarding LS-SNCs and U-LS-SNCs are illustrated in Figure 1. As can be observed in the figures, the ultrasonic treatment of LS helped shorten the acid hydrolysis time of the LS-SNCs. At day 1 of acid hydrolysis, the higher the ultrasonic power, the more obvious the aggregation of U-LS-SNC particles; at day 3 of acid hydrolysis, the higher the ultrasonic power, the more spherical crystals could be observed using U-LS-SNCs; at day 5 of acid hydrolysis, the higher the ultrasonic power, the more lamellar crystals can be observed using U-LS-SNCs. In Figure 1i pertaining to the LS-SNCs (0 W, 5 d), the surfaces of the massive aggregates were destroyed, and many square and spherical crystals appeared, but there were more numerous square crystals than the spherical crystals. However, as shown in Figure 1h, after ultrasonic treatment of the U-LS-SNCs (200 W, 3 d), small spherical crystals could be observed; compared with the preparation of LS-SNCs via acid hydrolysis for only 5 days, the preparation time of U-LS-SNCs was shortened by 2 days. This was because ultrasonic treatment destroyed the surface structure of the starch. The surfaces of the starch particles after ultrasonic treatment showed deformation and many small pits [27]. This was because of the damaged starch granule surface after the ultrasonic processing of LS. It was easier for sulfuric acid to enter the starch particles, and the many small pits increased the reaction area, thereby improving the reaction’s efficiency and shortening the reaction time. This result was consistent with those reported by Li et al. [28], who studied the effect of ultrasonic treatment on the structure and physicochemical properties of pea starch in an acid system.

### 3.2. Particle Sizes of LS-SNPs and U-LS-SNCs

**Figure 2:**
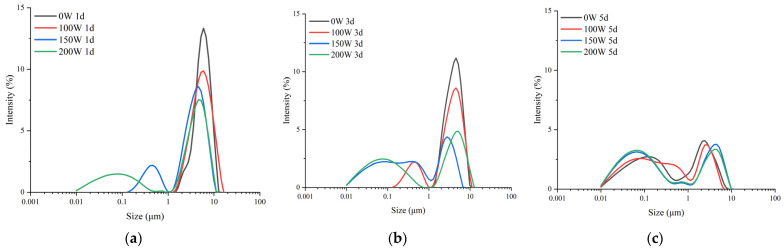
Particle size distribution of LS-SNCs prepared using acid hydrolysis and U-LS-SNCs prepared using ultrasonically assisted acid hydrolysis. U-LS-SNCs and LS-SNCs produced after treatment for (**a**) 1 d, (**b**) 3 d, and (**c**) 5 d.

Figure 2 shows the particle size distribution of the LS-SNCs and U-LS-SNCs. It can be seen from the figures that when the number of days of acid hydrolysis was the same, the particle size distribution decreased with the increase in ultrasonic power. After 1 day of acid hydrolysis, the U-LS-SNCs (150 W) and U-LS-SNCs (200 W) had a nanoscale particle size distribution; after 3 days of acid hydrolysis, the U-LS-SNCs (100 W) had a nanoscale particle size distribution, whereas the U-LS-SNCs (0 W) had a nanoscale particle size distribution after 5 days of acid hydrolysis. It can be seen that ultrasonic-assisted acid hydrolysis potentiated the reaction speed of acid hydrolysis and decreased the number of days of the acid hydrolysis reaction and that the efficiency of promoting the reaction increased with the increase in ultrasonic power. Shabana et al. [19] studied the structural characteristics of potato starch treated using ultrasonic-assisted acid hydrolysis, and the results showed that the particle size of potato starch treated via ultrasonic-assisted acid hydrolysis was smaller than that of potato starch treated via ultrasonic radiation alone and acid hydrolysis alone. The result in this paper is in line with these findings as SNCs with smaller particle sizes could be prepared using ultrasonic-assisted acid hydrolysis.

**Figure 3:**
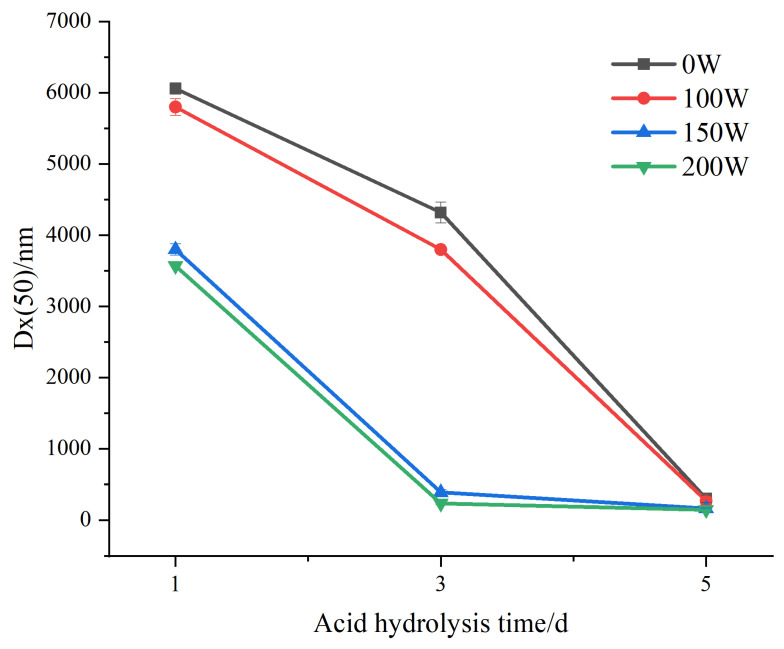
Change in Dx (50) of LS-SNCs prepared using acid hydrolysis and U-LS-SNCs prepared using ultrasonically assisted acid hydrolysis.

Figure 3 shows the change in the Dx (50) of the LS SNCs and U-LS SNCs with the increase in the number of acid hydrolysis days. Dx (50) refers to the corresponding particle size when the cumulative particle size distribution ratio of the SNCs exceeds 50%. The change trends of the particle size of the U-LS SNCs (150 W) and the U-LS SNCs (200 W) are similar. During 1–3 days of acid hydrolysis, the particle size of the U-LS SNCs (150 W) and U-LS SNCs (200 W) decreased relatively quickly. After reaching the nanometric level, the decline rate began to flatten (acid hydrolysis 3–5 d). The change trends regarding the particle size of the LS SNCs and U-LS SNCs (100 W) were also similar. The particle size decline rate during 1–3 days of acid hydrolysis was relatively gentle compared with that of the U-LS SNCs (150 W) and U-LS SNCs (200 W). However, during 3–5 days of acid hydrolysis, the particle size decline rate of the U-LS SNCs (150 W) and U-LS SNCs (200 W) continued to increase; finally, the particle size reached the nanometer level after 5 days of acid hydrolysis. The results show that when the ultrasonic power is less than 100 W, the promotion of acid hydrolysis is not obvious. When the ultrasonic power is 150–200 W, ultrasonication has a relatively strong role in promoting acid hydrolysis.

According to Table 1, under different ultrasonic power treatment conditions, when nanoscale particles first appeared, the D(50) values of the U-LS-SNCs (0 W, 5 d), U-LS-SNCs (100 W, 5 d), U-LS-SNCs (150 W, 3 d), and U-LS-SNCs (200 W, 3 d) were 302, 272, 392, and 237 nm, respectively. This shows that the preparation of SNCs only took 3 days via ultrasonic-assisted acid hydrolysis, which is 2 days shorter than that via acid hydrolysis alone. In addition, the D(50) values of the U-LS-SNCs (0 W, 5 d), U-LS-SNCs (100 W, 5 d), U-LS-SNCs (150 W, 5 d), and U-LS-SNCs (200 W, 5 d) were 302, 272, 166, and 147 nm, respectively. The results show that when the ultrasonic power was less than 200 W and the acid hydrolysis times were the same, the SNCs became smaller with the increase in ultrasonic power. This was because slight ultrasonic treatment only acted on the surface of the starch particles and did not damage their internal structure. However, excessive ultrasonic treatment would destroy the structure of starch particles and cause the particles with high surface activation energy to attract each other and aggregate, which are inconducive conditions for acid hydrolysis [29].

### 3.3. Mw Distribution

The overall *Mw* distributions of the LS-SNCs and U-LS-SNCs were assessed using gel permeation chromatography to investigate the effect of ultrasonic-assisted acid hydrolysis on *Mw*. Table 2 demonstrates that the *Mw* and the number-average molecular weight (*Mn*) of the U-LS-SNCs were smaller than those of the LS-SNCs and that the higher the ultrasonic power, the lower the *Mw* and the *Mn* of the U-LS-SNCs. The latter effect occurred because when the starch was pretreated using ultrasonic radiation, mechanical action and cavitation effects induced by ultrasonic radiation would lead to the fracture of the starch molecular chain and *Mw* reduction [30]. Therefore, acid hydrolysis after ultrasonic treatment can promote the reduction in the *Mw* and the *Mn* of SNCs. In addition, the degradation of natural dextrin using ultrasound mainly occurred in the macromolecular region, and the relative *Mw* degradation limited to natural dextrin via ultrasound was 4 × 10^4^ Da. The *Mw* and the *Mn* of the LS-SNCs and U-LS-SNCs in Table 2 were both less than 4 × 10^4^ Da, indicating that acid hydrolysis was still the main reason for the reduction in the weight-average molecular weight (*Mw*) of the starch particles.

In Table 2, the *Mw* distributions of the LS-SNCs and U-LS-SNCs showed no obvious differences at the same hydrolysis time, and the polydispersion index (*Mw/Mn*) in Table 2 indicates the range of *Mw* distributions: the *Mw/Mn* of all the LS-SNCs and U-LS-SNCs ranged from 1.73 to 2.15. The results show that ultrasonic treatment had little effect on the *Mw* distribution of the SNCs.

### 3.4. X-ray Diffraction

**Figure 4:**
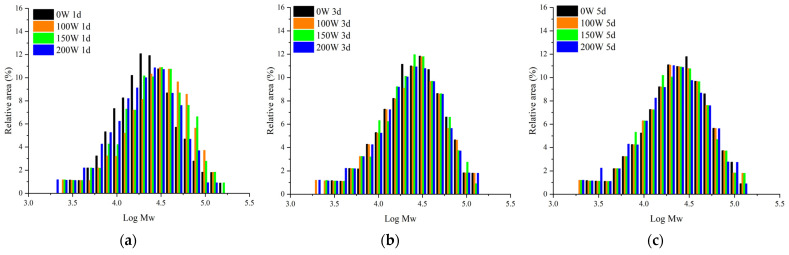
Molecular weight distribution of LS-SNCs prepared using acid hydrolysis and U-LS-SNCs prepared using ultrasonically assisted acid hydrolysis. U-LS-SNCs and LS-SNCs produced after treatment for (**a**) 1 d, (**b**) 3 d, and (**c**) 5 d.

The XRD patterns of the LS-SNCs and U-LS-SNCs are shown in Figure 4. The XRD patterns have diffraction peaks (2θ) at 15.00° and 22.93° and double peaks at 17.02° and 17.92°, corresponding to an A-type crystal structure. The XRD patterns have diffraction peaks (2θ) at 5.58°, 15.13°, 17.23°, 19.67°, 22.20°, 24.00°, and 26.35°, corresponding to a B-type crystal structure. A C-type crystal structure consists of A-type and B-type crystals. The LS-SNCs and U-LS-SNCs had characteristic diffraction peaks at 2θ = 15.1° and 23.1°, double peaks at 2θ = 17.1° and 18.1°, and weak peaks at 2θ = 11.2° and 26.4°, corresponding to both A-type and B-type characteristics, indicating that the LS-SNCs and U-LS-SNCs all had a C-type crystal structure. The results are in line with those reported by Singh et al. [31], who found that ultrasonic pretreatment did not change the crystalline structure of starch.

**Figure 5:**
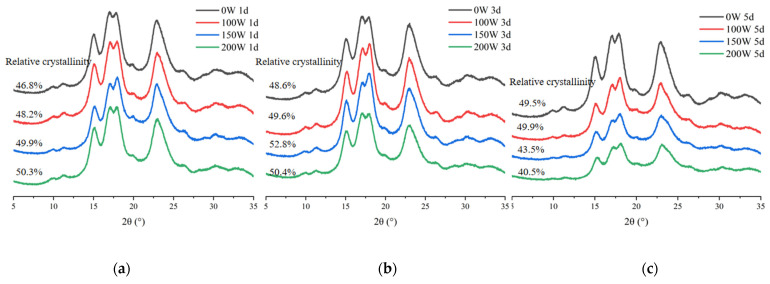
X-ray diffraction patterns of LS-SNCs prepared using acid hydrolysis and U-LS-SNCs prepared using ultrasonically assisted acid hydrolysis. U-LS-SNCs and LS-SNCs produced after treatment for (**a**) 1 d, (**b**) 3 d, and (**c**) 5 d.

The calculation of relative crystallinity was performed according to the method reported Dome et al. [26]. The results show that the relative crystallinity of the U-LS-SNCs could be improved by increasing the ultrasonic power after day 1 of acid hydrolysis; the relative crystallinity of the U-LS-SNCs increased first and then decreased with the increase in ultrasonic power at days 3 and 5 of acid hydrolysis; and the maximum relative crystallinity of U-LS-SNCs (150 W, 3 d) was 52.8%. The crystal region of LS had a compact structure, and ultrasonic cavitation and high-frequency oscillation mainly acted on the amorphous region of LS. Thus, ultrasonic radiation can promote acid hydrolysis. The main reason for the decrease in the relative crystallinity of the U-LS-SNCs prepared at 150 W and 200 W ultrasonic power values was acid hydrolysis, which accelerated after the amorphous region was destroyed by ultrasonic radiation [32]. Finally, the sharpness of the peaks in the XRD pattern could also indicate a change in the crystal region, for which the more obvious and sharper the peaks, the more complete the crystal region. The change in the peaks in Figure 5 is in line with the change trend of relative crystallinity.

### 3.5. FT-IR Spectroscopy

**Figure 6:**
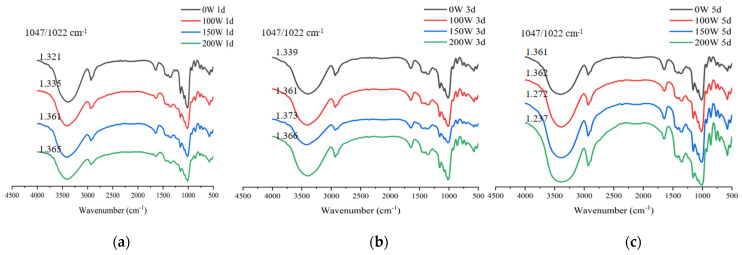
FT-IR diagram of LS-SNCs prepared using acid hydrolysis and U-LS-SNCs prepared using ultrasonically assisted acid hydrolysis. U-LS-SNCs and LS-SNCs produced after treatment for (**a**) 1 d, (**b**) 3 d, and (**c**) 5 d.

The FT-IR spectra of the LS-SNCs and U-LS-SNCs are presented in Figure 6. In Figure 5, it can be seen that the LS-SNCs and U-LS-SNCs had similar FT-IR spectra, indicating that the main structure of the starch particles was not damaged by ultrasonic radiation. This result is consistent with the results reported by Pourmohammadi et al. [33]. In the FT-IR spectrum of starch, the peak intensity ratio of 1045 cm^–1^/1022 cm^–1^ was a parameter used to measure the ordered structure of starch, for which the larger the ratio, the higher the degree of order [34]. The maximum value of 1045 cm^–1^/1022 cm^–1^ of the U-LS-SNCs (150 W, 3 d) corresponded to 1.373, and under this condition, the relative crystallinity was also the highest; thus, the two results are in accordance.

## 4. Conclusions

In this study, the SEM data, particle size, molecular weight, *Mw* distribution, XRD patterns, and FT-IR spectra of LS-SNCs and U-LS-SNCs were compared. Compared with the preparation method for the LS-SNCs, the U-LS-SNCs had the advantage of a shorter preparation time, which was shortened from five days to three days. Compared with the structural characteristics of the LS-SNCs, the surface of the starch particles was destroyed after ultrasonic pretreatment, which increased the reaction area between starch and sulfuric acid and allowed the sulfuric acid to enter the starch particles and promote the acid hydrolysis reaction. Therefore, the U-LS-SNCs (200 W, 5 d) had the smallest particle size and *Mw*. Ultrasonic treatment did not change the crystalline structure and composition structure of the starch particles, but ultrasonic treatment accelerated the acid hydrolysis speed after the destruction of the amorphous region. Therefore, the higher the ultrasonic power and the more days of acid hydrolysis, the greater the relative crystallinity of SNCs was reduced. The U-LS-SNCs (150 W, 3 d) presented the maximum relative crystallinity. This study provides a novel concept for the preparation of SNCs. Reducing SNCs’ particle size is conducive to the preparation of a stabler Pickering emulsion [35]. SNCs have good barrier properties. As food packaging materials, SNCs can improve the water vapor permeability of edible films [36]. In addition, starch nanocrystals are often used as fillers because of their high modulus, high strength, good biocompatibility, degradability, renewability, and other characteristics that are not offered by inorganic nanoparticles [37]. Furthermore, the improvement of the structural characteristics of SNCs will promote the application of SNCs in food processing.

## Figures and Tables

**Table 1:** Particle size distribution of LS-SNCs prepared using acid hydrolysis and U-LS-SNCs prepared using ultrasonically assisted acid hydrolysis.

Acid Hydrolysis Days/d	Ultrasonic Power/W	D [3,2]/nm	D [4,3]/nm	Dx (10)/nm	Dx (50)/nm	Dx (90)/nm
1	0	5370 ± 60.3 ^a^	6280 ± 86.2 ^a^	3420 ± 55.1 ^a^	6060 ± 80.8 ^a^	9480 ± 128.6 ^b^
	100	5040 ± 100.0 ^b^	6330 ± 125.0 ^a^	2920 ± 60.3 ^b^	5800 ± 115.0 ^b^	10,600 ± 200.0 ^a^
	150	1510 ± 63.5 ^c^	3940 ± 92.4 ^b^	470 ± 16.7 ^c^	3800 ± 80.8 ^c^	7310 ± 138.6 ^c^
	200	170 ± 7.6 ^d^	3560 ± 1.7 ^c^	49.7 ± 1.5 ^d^	3570 ± 0.6 ^d^	7480 ± 1.0 ^c^
3	0	3870 ± 105.4 ^a^	4580 ± 141.9 ^a^	2370 ± 50.3 ^a^	4320 ± 145.7 ^a^	7250 ± 218.3 ^a^
	100	1510 ± 63.5 ^b^	3940 ± 92.4 ^b^	470 ± 16.7 ^b^	3800 ± 0.8 ^b^	7310 ± 138.6 ^a^
	150	109 ± 4.7 ^c^	1310 ± 55.1 ^c^	35.6 ± 1.3 ^c^	392 ± 38.7 ^c^	3850 ± 79.4 ^b^
	200	88.7 ± 1.3 ^c^	1050 ± 69.3 ^d^	29.6 ± 0.3 ^c^	237 ± 13.0 ^d^	6030 ± 133.2 ^c^
5	0	107 ± 4.9 ^a^	1280 ± 167 ^bc^	36.4 ± 1.2 ^a^	302 ± 49.9 ^a^	3700 ± 443.8 ^b^
	100	94.3 ± 6.6 ^b^	1070 ± 115.8 ^c^	31.9 ± 1.6 ^b^	272 ± 53.5 ^a^	3390 ± 245.0 ^b^
	150	79 ± 1.3 ^c^	1660 ± 40.0 ^a^	27.5 ± 0.3 ^c^	166 ± 7.6 ^b^	5390 ± 37.9 ^a^
	200	75.3 ± 0.2 ^c^	1500 ± 173.2 ^ab^	26.7 ± 0.1 ^c^	147 ± 4.6 ^b^	5120 ± 10.0 ^a^

Data are the averages of three determinations. Different letters in the same column indicate significant differences (*p* < 0.05).

**Table 2:** Molecular weight distribution of LS-SNCs prepared using acid hydrolysis and U-LS-SNCs prepared using ultrasonically assisted acid hydrolysis.

Acid Hydrolysis Days/d	Ultrasonic Power/W	*Mw* (×10^4^ Da)	*Mn* (×10^4^ Da)	*Mw/Mn*
1	0	4.3830	2.5387	1.7265
	100	4.1468	1.9667	2.1085
	150	4.1307	1.9490	2.1194
	200	4.0334	1.8858	2.1388
3	0	3.8200	2.0957	1.8228
	100	3.7195	1.7706	2.1007
	150	3.6718	1.7470	2.1018
	200	3.5768	1.6661	2.1468
5	0	3.5936	1.9048	1.8866
	100	3.5684	1.7653	2.0214
	150	3.5002	1.7083	2.0489
	200	3.4179	1.5933	2.1452

## Data Availability

The datasets used and analyzed during the current study are available from the corresponding author on reasonable request.
